# Circulating *ERVFRD-1* and *MFSD2A* Are Associated with Immunotherapy Response in Metastatic Clear Cell Renal Cell Carcinoma

**DOI:** 10.3390/cancers18040716

**Published:** 2026-02-23

**Authors:** Hector Katifelis, Styliani-Evangelia Zerva, Aristotelis Bamias, Michalis V. Karamouzis, Konstantinos Stravodimos, Leonardo A. Sechi, Dimitra-Ioanna Lampropoulou, Evangelia Pliakou, Maria Gazouli

**Affiliations:** 1 Laboratory of Biology, Department of Basic Medical Sciences, Medical School, National and Kapodistrian University of Athens, Michalakopoulou 176, 11527 Athens, Greece; katifel@med.uoa.gr (H.K.); stellazerva03@gmail.com (S.-E.Z.); 2 2nd Propaedeutic Department of Internal Medicine, ATTIKON University Hospital, School of Medicine, National and Kapodistrian University of Athens, 11527 Athens, Greece; abamias@med.uoa.gr; 3 Molecular Oncology Unit, Department of Biological Chemistry, National and Kapodistrian University of Athens, 11527 Athens, Greece; mkaramouz@med.uoa.gr; 4 Department of Urology, National and Kapodistrian University of Athens, “Laiko” Hospital, 11527 Athens, Greece; kstravd@med.uoa.gr; 5 Department of Biomedical Sciences, University of Sassari, 07100 Sassari, Italy; sechila@uniss.it; 6 Department of Nutritional Science and Dietetics, Faculty of Health Sciences, University of Peloponnese, 24100 Kalamata, Greece; d.lampropoulou@uop.gr; 7 Department of Medical Oncology, Athens Naval and Veterans Hospital, 11521 Athens, Greece; evangeliaplk@hotmail.com

**Keywords:** ccRCC, immunotherapy, ICIs, PD-1, TKI, *MFSD2A*, *ERVFRD-1*

## Abstract

**Background/Objectives**: Immune checkpoint inhibitor (ICI)-based combinations have significantly improved outcomes in metastatic clear cell renal cell carcinoma (mccRCC). However, a substantial number of patients fail to derive clinical benefit, highlighting the need for reliable predictive biomarkers. Circulating biomarkers represent an attractive, non-invasive alternative to tissue-based assays. This study aimed to evaluate the immunity-related genes *ERVFRD-1* and *MFSD2A* as potential blood-based candidate biomarkers associated with response to ICI-based therapy in mccRCC. **Methods**: Peripheral blood samples were collected prior to treatment initiation from 34 patients with mccRCC receiving PD-1-based therapy. Gene expression levels of *ERVFRD-1* and *MFSD2A* were quantified using real-time PCR. Treatment response was assessed according to the Response Evaluation Criteria in Solid Tumors (RECIST) version 1.1. Peripheral blood samples from healthy individuals were included as controls. **Results**: Both *ERVFRD-1* and *MFSD2A* were significantly dysregulated in mccRCC patients compared with healthy controls. Their expression differed between patients with clinical benefit and those with progressive disease. Specifically, patients with progressive disease exhibited reduced *ERVFRD-1* expression and increased *MFSD2A* expression compared with patients showing clinical benefit. **Conclusions**: *ERVFRD-1* and *MFSD2A* were associated with treatment response in this pilot cohort and may represent promising blood-based biomarker candidates, requiring validation in larger prospective multicenter studies.

## Simple Summary

Immune checkpoint inhibitor (ICI) therapies have improved outcomes for patients with metastatic clear cell renal cell carcinoma (mccRCC). However, many patients do not respond to treatment. Therefore, reliable biomarkers associated with therapeutic response are urgently needed. Blood-based biomarkers offer a non-invasive alternative to tissue analysis. In this study, we investigated the expression of two immunity-related genes, *ERVFRD-1* and *MFSD2A*, in peripheral blood samples from mccRCC patients receiving PD-1-based treatment. Both genes were dysregulated compared with healthy controls and demonstrated differential baseline expression between patients who achieved clinical benefit and those with progressive disease. Patients with progressive disease exhibited decreased expression of *ERVFRD-1* and increased expression of *MFSD2A*. These findings suggest that *ERVFRD-1* and *MFSD2A* may serve as candidate blood-based biomarkers associated with response to ICI, although confirmation in larger prospective studies is required.

## 1. Introduction

Renal cell carcinoma (RCC) represents a heterogeneous malignancy with a rising global incidence, accounting for more than 80% of all kidney cancers. Recent data further indicate that RCC represents approximately 4% of all new cancer cases [1,2]. The most common histological subtype of RCC is clear cell RCC (ccRCC), which accounts for approximately 75% of renal malignancies, followed by papillary and chromophobe tumors [3]. Unfortunately, ccRCC tends to be highly metastatic (metastatic ccRCC, mccRCC). Nearly one out of three patients present with distant metastases (including bone, brain, and pancreas) at the time of diagnosis [4,5].

Immune-checkpoint inhibitors (ICIs) have drastically improved the outcomes of mccRCC patients over the past years [6]. These agents are used in combinations that typically include programmed cell death-1 (PD-1) inhibitors combined with another type of ICI, namely cytotoxic T-lymphocyte-associated protein 4 (CTLA-4) inhibitors. Alternatively, PD-1 inhibitors can be used in combination with a tyrosine kinase inhibitor (TKI). Both combinations are recommended by the European Society of Medical Oncology (ESMO) [7] and the American Society of Clinical Oncology (ASCO) [8].

However, despite these advances, approximately 40–50% of mccRCC patients fail to derive durable benefit from ICI/ICI or ICI/TKI combinations, highlighting a critical unmet clinical need for robust predictive biomarkers [9,10]. It remains a major challenge for personalized oncology approaches to identify these patients, and the optimal predictive biomarker remains to be established. The first biomarker to be used for this purpose was PD-L1. Its expression has been associated with immunotherapy response in several cancers, but its use has been problematic in several malignancies, including mccRCC [11,12]. From a clinical perspective, repeated tumor biopsies in patients with advanced disease are often impractical or unsafe. Therefore, circulating biomarkers represent a particularly attractive, minimally invasive alternative with increasing clinical relevance [13,14,15].

Among circulating candidates, genes involved in immune regulation and interactions between tumor and immune components have attracted increasing attention, particularly those with established roles in immune barrier integrity and immune tolerance [14,15]. In this context, two genes of particular importance are *ERVFRD-1* (Endogenous Retrovirus Group FRD member 1) [16] and *MFSD2A* (Major Facilitator Superfamily Domain Containing 2A) [17]. *ERVFRD-1* encodes Syncytin-2, which engages *MFSD2A* as its cognate receptor, forming a defined ligand–receptor pair in human placentation, and has been shown to possess immunosuppressive properties that have been proposed to contribute to immune tolerance mechanisms [18,19].

In addition to its physiological role in placentation and maternal immune tolerance [16], *ERVFRD-1* has also been implicated in tumor–immune interactions. A recent comprehensive bioinformatic analysis by Wen et al. [16] demonstrated that *ERVFRD-1* expression in ccRCC tumor tissue is significantly associated with immune-related pathways, tumor mutation burden, and patient survival, proposing *ERVFRD-1* as a promising biomarker for prognosis and immunotherapy response. Importantly, however, evidence regarding its potential utility as a circulating biomarker remains extremely limited. *ERVFRD-1* promotes the inhibition of anti-tumor immune responses in experimental models and has been shown to influence tumor growth in human cancer cells. Conversely, in the context of RCC, reduced expression has been reported, while higher expression appears to correlate with improved survival [16,20]. Furthermore, the current literature suggests that it acts as a regulator of the tumor microenvironment in patients with melanoma [16].

Beyond its role as the Syncytin-2 receptor, *MFSD2A* has been implicated in several processes relevant to immune regulation and tumor biology [21]. This gene encodes a protein involved in lipid metabolism with a major role in the maintenance of the blood–brain barrier (BBB) and its deficiency is linked to BBB leakage and developmental disorders [22]. At the same time, it represents an important component of CD8 memory T cell function participating in both T cell maintenance and immune response [23]. In cancer, it has been described as a tumor suppressor in hepatocellular carcinoma and lung cancer [24,25]. Its overexpression has been found to repress cellular proliferation and migration in vitro and tumor growth in vivo [26]. Interestingly, it has been studied as a predictive biomarker for anti-PD-1 immunotherapy in gastric cancer [17]. In addition, Shi et al. [27] reported that the expression of *MFSD2A* is reduced in gastric tumors while increased expression was associated with improved survival and clinicopathologic features consistent with a less aggressive phenotype. These findings support the tumor-suppressive role of *MFSD2A* in the context of this malignancy.

In our previous work [28], we investigated the potential of the expression of circulating inflammation- and immunity-related genes as biomarkers of immunotherapy effectiveness in mccRCC individuals. In the present study, we aimed to further explore the potential of the expression of the immunity-related genes *MFSD2A* and *ERVFRD-1* as candidate biomarkers associated with response to ICI-based therapies in mccRCC. To our knowledge, this is the first study investigating the association between circulating *ERVFRD-1* and *MFSD2A* expression levels and response to ICI-based therapies in metastatic clear cell renal cell carcinoma.

## 2. Materials and Methods

### 2.1. Patients

Peripheral blood was obtained from 34 patients diagnosed with mccRCC before the start of therapy. All patients received first-line anti-PD-1 blockade (Nivolumab or Pembrolizumab) combined with either CTLA-4 inhibition (Ipilimumab) or a TKI (Cabozantinib or Axitinib) for metastatic disease.

Treatment outcomes were grouped by clinical benefit (CB) [comprising complete response (CR), partial response (PR), and stable disease, (SD)] or progressive disease (PD). Response categorization was evaluated according to RECIST version 1.1 criteria [29].

Patients were managed at Attikon University Hospital and Laikon General Hospital during the period between 2021 and 2023. Blood samples from 30 healthy donors served as the control group. Written informed consent was obtained from all individuals prior to inclusion, and the protocol received approval from the ethics committees of both institutions. All samples were collected and processed according to a standardized workflow across participating centers. The time from collection to processing was minimized and samples were handled under uniform temperature conditions. Repeated freeze–thaw cycles were avoided. Duplicate measurements differing by more than one Ct cycle were considered unreliable and were excluded from further analysis.

### 2.2. RNA Extraction and cDNA Synthesis

Total RNA was isolated from whole blood using the Qiagen AllPrep RNA/DNA Mini Kit (Qiagen, Hilden, Germany) according to the manufacturer’s instructions. Complementary DNA (cDNA) was generated from the extracted RNA with a TAKARA kit (Takara Bio Europe SAS, Saint-Germain-en-Laye, France). Reverse transcription was carried out in a SuperCycler Thermal Cycler (Kyratec, Mansfield, Australia) at 37 °C for 30 min, followed by 5 min at 85 °C to deactivate reverse transcriptase.

### 2.3. Real-Time PCR and Gene Expression Analysis

Real-time PCR was performed using KAPA SYBR FAST qPCR mix (KAPA BIOSYSTEMS, Cape Town, South Africa). *GAPDH* served as a reference gene, and primer sequences for *ERVFRD-1*, *MFSD2A* and *GAPDH* are listed in Table 1.

Each sample was analyzed in duplicate, and relative expression levels were calculated after normalization to *GAPDH*. Amplification reactions were run on a SaCycler-96 (Sacace Biotechnologies, Como, Italy). Changes in transcript levels were determined using the 2^−ΔΔCt^ is presented as fold regulation. Gene downregulation is shown as the negative inverse of fold change and overexpressed genes are shown as fold change [30]. All RT-qPCR reactions were performed in technical duplicates. Intra-assay variability was assessed using Ct values, with coefficients of variation consistently below 5%. Duplicate measurements differing by more than one Ct cycle were excluded from analysis.

### 2.4. Statistical Analysis

Data analysis was conducted with GraphPad Prism (version 10.6.1; GraphPad Software, San Diego, CA, USA). Statistical significance was defined as a *p*-value < 0.05.

## 3. Results

### 3.1. Patient Characteristics, Treatment and Clinical Response

The study cohort consisted of 34 patients with males representing the majority (*n* = 26). The mean age at treatment initiation was 67.1 ± 10.6 years. Regarding treatment regimens, 16 patients (47%) received nivolumab plus Ipilimumab as first-line treatment. Nine patients (26.5%) received nivolumab plus Cabozantinib whereas another nine patients (26.5%) received Pembrolizumab plus Axitinib. Clinical benefit was observed for 53% of patients, while 47% showed PD. A summary of the study cohort is presented in Table 2.

### 3.2. ERVFRD-1 and MFSD2A Are Downregulated in mccRCC Patients

**Figure 1:**
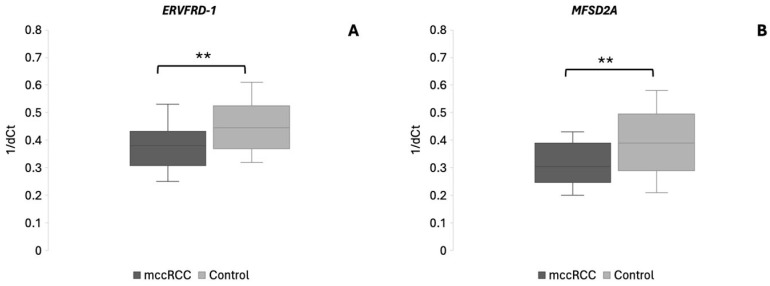
Differentially expressed genes *ERVFRD-1* (panel (**A**)) and *MFSD2A* (panel (**B**)) between mccRCC patients and healthy individuals. Data are shown as boxplots with the y axis representing 1/dCt values. Median 1/dCt values of each group are shown with a horizontal line. ** *p* < 0.01.

The expression of *MFSD2A* and *ERVFRD-1* was evaluated among mccRCC patients and healthy individuals. Both genes exhibited significantly decreased expression in mccRCC patients compared with healthy individuals, suggesting systemic dysregulation of immune-related pathways in this disease. *ERVFRD-1* was significantly downregulated in mccRCC patients (0.55-fold change, *p* = 0.002), while *MFSD2A* was similarly reduced (0.65-fold change, *p* = 0.0021), supporting the biological relevance of these circulating transcripts in the context of disease presence. The results are shown in Figure 1 (panel A for *MFSD2A* and panel B for *ERVFRD-1*).

### 3.3. Transcriptomic Differences Between CB and PD Groups at Baseline

**Figure 2:**
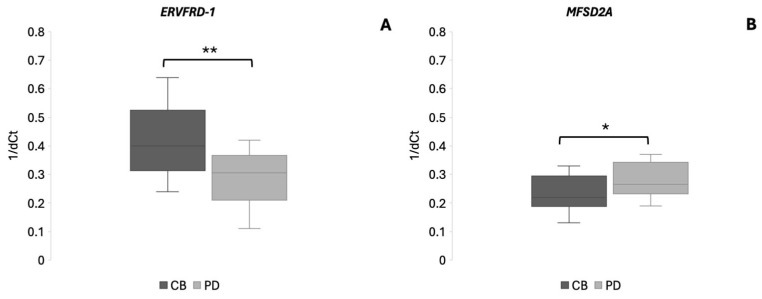
Differentially expressed genes *ERVFRD-1* (panel (**A**)) and *MFSD2A* (panel (**B**)) among mccRCC patients in CB and PD groups. Data are shown as boxplots with the y axis representing 1/dCt values. Median 1/dCt values of each group are shown with a horizontal line. * *p* < 0.05; ** *p* < 0.01.

To evaluate potential differences in *ERVFRD-1* and *MFSD2A* expression between PD and CB groups we investigated gene expression across these patient groups. Regarding *ERVFRD-1*, compared to PD patients, the CB group showed increased expression by approximately 1.4-fold (*p* = 0.0014). Conversely, *MFSD2A* showed a modest but statistically significant increase in PD patients compared with the CB group (1.2-fold, *p* = 0.044). Further subgroup analyses based on specific treatment regimens were not feasible due to small sample sizes. The results are shown in Figure 2.

## 4. Discussion

The use of ICI-based approaches has been revolutionary in cancer therapeutics; nevertheless, only a subset of mccRCC patients benefits from these treatments [31]. Thus, the need for predictive biomarkers to achieve personalized oncology approaches is urgent. Extending our previous work [28], we investigated potential differences in the expression of the immunity-related genes *MFSD2A* and *ERVFRD-1* between CB and PD patients.

Regarding *ERVFRD-1* and other members of the *HERV* family, there is a growing body of evidence that they are dysregulated in several malignancies, including ccRCC, prostate and bladder cancer [16,32,33]. Indeed, our results show that *ERVFRD-1* is downregulated in the blood of mccRCC patients. To the best of our knowledge, this is the first study to evaluate *ERVFRD-1* expression in the peripheral blood of mccRCC patients. A recent large-scale bioinformatic analysis demonstrated that *ERVFRD-1* expression correlates with immune activation signatures, tumor mutation burden, and overall survival in ccRCC, supporting a biologically plausible link between *ERVFRD-1* and immunotherapy responsiveness [16]. Although the results shown by Wen et al. did not show a statistically significant association with response to PD-L1/PD-1 blockade, this analysis was performed in a retrospective subset comprising a limited number of treated patients and was based on tumor tissue. Nevertheless, the authors suggested an important role of *ERVFRD-1* in immune activation mediating an anti-tumor effect in ccRCC and found that higher *ERVFRD-1* expression is associated with progression-free survival.

In line with these observations, *ERVFRD-1* (Syncytin-2) expression in our cohort was downregulated in PD patients, whereas higher expression levels were observed in CB patients. Taken together, these findings, combined with our observation that higher circulating *ERVFRD-1* expression is associated with clinical benefit, support the investigation of *ERVFRD-1* as a potential blood-based biomarker for patient stratification in mccRCC.

At first glance, this observation seems to contrast with the concept of viral mimicry, according to which derepression of epigenetically silenced ERVs generates double-stranded RNA that activates innate sensors and induces interferon-driven antitumor immunity [34,35]. However, circulating RNA levels do not necessarily mirror intratumoral ERV activity and are more likely to represent a systemic immune context shaped by host inflammatory status and immune cell composition [15]. We therefore hypothesize that reduced peripheral *ERVFRD-1* expression may indicate weakened interferon pathway activation and impaired cytotoxic competence. Such defects have been repeatedly associated with reduced responsiveness to ICIs [36,37], offering a biologically plausible explanation for the inverse relationship observed in our cohort. Additionally, Syncytin-2 possesses immunomodulatory properties and is subject to epigenetic regulation [16].

From an evolutionary perspective, Syncytin-2 and its receptor (MFSD2A) represent typical examples of endogenous retroviral exaptation, where ancestral viral envelope genes were co-opted to enable placentation and maternal-fetal immune tolerance [18,38]. During pregnancy, trophoblast cells deploy coordinated immune-evasion strategies (including local suppression of cytotoxic responses) to prevent immune rejection of the semi-allogenic fetus. Several of these mechanisms are similar to strategies exploited by tumors to achieve evasion of host immunity [39]. Consequently, altered *ERVFRD-1* and *MFSD2A* expression in cancer may indicate activation of tolerance-like mechanisms within the tumor-host interaction and not intrinsic tumor immunogenicity per se. This evolutionary parallel provides a conceptual framework for interpreting why expression patterns associated with immunity regulation in pregnancy may influence ICI responsiveness. It further suggests that characterization of placenta-derived tolerance pathways in tumors could refine patient stratification and could identify therapeutic opportunities aiming at reversing immune permissiveness, for example through combinations of ICIs with agents that modulate epigenetic or interferon-related mechanisms. Nevertheless, future studies that integrate tumor tissue analyses with peripheral measurements are necessary to clarify compartment-specific effects.

In other malignancies, such as acute myeloid leukemia, increased Syncytin-2 levels indicate a favorable prognosis in particular subgroups, including patients with low white blood count and those of white race [40]. However, its exact role in immunotherapy response remains veiled. Interestingly, *ERVFRD-1* has been proposed as a therapeutic target to enhance immunotherapy efficacy [34], with hypomethylating agents such as decitabine potentially sensitizing tumors to immunotherapy. Indeed, methylation has been shown [16] to play a key role in *ERVFRD-1* regulation with increased methylation associated with an unfavorable prognosis.

*MFSD2A* has also been implicated in multiple cancers. Loss of *PRMT6* (a gene encoding a methyltransferase) induces *MFSD2A* upregulation, promoting leukemia stem cells maintenance [41]. While *MFSD2A* has been investigated in tumor tissue across several malignancies, data regarding its circulating expression in mccRCC are currently lacking. In our cohort, *MFSD2A* was significantly downregulated in peripheral blood of mccRCC patients compared with healthy individuals. Interestingly, Wang et al. [42] showed that ccRCC, *MFSD2A* and *CYP24A1* were positively correlated with risk scores in bioinformatic analyses, and patients in the high-risk group treated for advanced RCC showed lower half-maximum inhibition concentration of targeted therapeutic agents (Sunitinib, Temsirolimus and Pazopanib).

In our study, *MFSD2A* was found to be upregulated in PD patients. Although the therapeutic contexts differ, these findings suggest that *MFSD2A* upregulation may be associated with unfavorable outcomes, such as a high-risk score or PD. Interestingly, *MFSD2A* has been shown to enhance response to PD-1 blockade in gastric cancer by reprogramming the tumor microenvironment and promoting cytotoxic T-cell activation [17]. The contrasting directionality observed in our cohort underscores the likelihood that *MFSD2A* exerts tumor-specific immunobiological functions, warranting further mechanistic investigation [17]. The favorable prognostic association of higher *MFSD2A* expression observed in gastric cancer [27] further supports that the biological role of this molecule could be highly context dependent. One possible explanation for this discrepancy may lie in the fundamental differences in tumor microenvironment, immune contexture and therapeutic exposure between gastric cancer and mccRCC. Clear cell renal cell carcinoma is widely recognized as an immunogenic malignancy characterized by dense immune infiltration and strong tumor-host immune interplay, features that influence responsiveness to ICIs [43]. In contrast, gastric cancer has traditionally been managed with chemotherapy-based treatments and the immune landscape driving treatment sensitivity may substantially differ [44]. Thus, the biological consequences of *MFSD2A* expression may substantially vary across tumor types, reflecting distinct immune escape mechanisms and microenvironmental pressures. While in gastric malignancies its increased expression has been linked to reduced angiogenesis and a less aggressive phenotype, our findings in mccRCC derive from circulating RNA levels in patients who received ICIs. These important differences in tumor type, sampling compartment and therapeutic context are possibly major contributors to these divergent observations. Overall, this variability highlights the presence of tumor-specific biological mechanisms that mediate response to immunotherapy.

Taken together, both genes show statistically significant differences between CB and PD groups supporting their potential as candidate biomarkers in this pilot cohort. Nevertheless, the interaction between *ERVFRD-1* (Syncytin-2) and *MFSD2A* in immunotherapy response or resistance remains largely unexplored.

This study has some limitations. The cohort size was limited, reflecting the difficulty of assembling uniformly treated mccRCC populations in real-world settings. This challenge is further complicated by the different therapeutic combinations (ICI/ICI or ICI/TKI) which limit the numbers of patients in each group. Given the exploratory nature of the study and the modest cohort size, no predictive model was constructed and internal validation approaches such as cross-validation or bootstrap resampling were not applied; these analyses will be essential in future larger cohorts aimed at model development and clinical prediction. While standardized sample handling and duplicate RT-qPCR measurements were implemented to enhance analytical robustness, only a single baseline blood sample per patient was available. As a result, intra-patient temporal variability and short-term biological fluctuations of *ERVFRD-1* and *MFSD2A* expression prior to treatment initiation could not be formally assessed. Consequently, it remains unclear whether the observed low or high circulating expression levels represent stable systemic phenotypes or dynamic transcriptional states. In addition, relevant clinical variables that may influence circulating RNA expression, such as systemic inflammatory status (e.g., neutrophil-to-lymphocyte ratio), tumor burden, performance status, and prior anti-angiogenic therapy, were not uniformly available and could not be included in correlation analyses or multivariable adjustment. Therefore, the independence of the observed associations from these potential confounders cannot be established in the present cohort. Intra-assay reproducibility was high, with coefficients of variation for Ct values consistently below 5%; however, longitudinal sampling will be required to establish biological coefficients of variation and transcript stability over time.

From a biological perspective, circulating gene expression may not fully reflect the tumor microenvironment. Importantly, the present study was not designed to establish causal or mechanistic relationships between circulating *ERVFRD-1* and *MFSD2A* expression and immunotherapy response. Therefore, the observed associations should be interpreted as hypothesis-generating rather than mechanistically explanatory. Functional studies, as well as paired tumor–blood and longitudinal analyses, will be necessary to clarify the biological mechanisms underlying these observations. Technical and pre-analytical considerations should also be acknowledged, as circulating RNA measurements are inherently sensitive to pre-analytical and biological variability. The dichotomization of patients using cohort-derived cut-offs was performed for exploratory purposes and does not imply the existence of clinically validated thresholds. Establishment of fixed reference ranges for circulating *ERVFRD-1* and *MFSD2A* will require assay harmonization and evaluation across multiple independent populations, including healthy controls. While standardized handling and duplicate real-time PCR measurements were implemented to enhance robustness, only a single baseline sample per patient was available and intra-patient temporal variability could not be assessed. Confirmation of transcript stability via serial sampling will be required before routine clinical application can be considered. From an analytical perspective, establishment of clinically applicable thresholds will require assay harmonization and the definition of validated reference ranges across independent populations. In addition, detailed time-to-event follow-up data were not uniformly available in this cohort. Therefore, survival analyses such as Kaplan–Meier estimation were not performed. Important potential confounders, including systemic inflammatory indices and tumor burden measurements were not uniformly available, precluding formal multivariable adjustment.

Finally, this was a two-center cohort from a single country. Thus, larger prospective studies are needed to validate the predictive value of *MFSD2A* and *ERVFRD-1.* The cut-off values applied for group comparisons were derived from the distribution of the present cohort and should be considered exploratory. Taken together, these considerations emphasize the need for external validation while supporting the role of the present findings as hypothesis-generating.

## 5. Conclusions

Circulating *ERVFRD-1* and *MFSD2A* expression showed an association with response to ICI-based immunotherapy in mccRCC patients. Downregulated *ERVFRD-1* expression and upregulated *MFSD2A* expression were associated with progressive disease. Collectively, our findings suggest that circulating *ERVFRD-1* and *MFSD2A* expression profiles represent non-invasive candidate biomarkers associated with response to ICI-based therapy in mccRCC. If validated in larger prospective cohorts, these biomarkers could contribute to improved patient stratification and support the implementation of precision immuno-oncology in routine clinical practice. Given the inherent sensitivity of circulating RNA measurements to pre-analytical and biological variability, confirmation of these observations with longitudinal sampling and standardized workflow will be essential prior to routine clinical implementation. Therefore, these results should be considered hypothesis-generating and interpreted in the context of the modest sample size, requiring independent validation.

## Figures and Tables

**Table 1:** Primer sequences.

Gene	Forward Primer	Reverse Primer
*ERVFRD-1*	5′-CCCTCACCCC CTTATTTCAT-3′	5′-TTTGAAGGACTA CGGCTGCT-3′
*MFSD2A*	5′-ATCAGCACCGAGCAGACTG-3′	5′-GCTATTGAGGTCCTGGAAACAAG-3′
*GAPDH*	5′-AGGTGGTCT CCTCTGACT TC-3′	5′-CTGTTGCTGTAGCCAAAT TCG-3′

**Table 2:** Patient clinicopathological data.

Characteristic		Number of Patients (%)
Gender	Male Female	26 (76%) 8 (24%)
Average age (Range)		67.1 ± 10.6 years
Treatment	Nivolumab + Ipilimumab Nivolumab + Cabozantinib Pembrolizumab + Axitinib	16 (47%) 9 (26.5%) 9 (26.5%)
Response Status	Clinical Benefit Progressive Disease	18 (53%) 16 (47%)

## Data Availability

The data generated and/or analyzed during the current study are available from the corresponding author on reasonable request.
